# Vision-Based Novelty Detection Using Deep Features and Evolved Novelty Filters for Specific Robotic Exploration and Inspection Tasks

**DOI:** 10.3390/s19132965

**Published:** 2019-07-05

**Authors:** Marco Antonio Contreras-Cruz, Juan Pablo Ramirez-Paredes, Uriel Haile Hernandez-Belmonte, Victor Ayala-Ramirez

**Affiliations:** 1 Department of Electronics Engineering, University of Guanajuato, Campus Irapuato-Salamanca, Carr. Salamanca-Valle de Santiago Km 3.5 + 1.8, Comunidad de Palo Blanco, Salamanca 36885, Mexico; 2 Department of Art and Enterprise, University of Guanajuato, Campus Irapuato-Salamanca, Carr. Salamanca-Valle de Santiago Km 3.5 + 1.8, Comunidad de Palo Blanco, Salamanca 36885, Mexico

**Keywords:** visual inspection, one-class classifier, grow-when-required neural network, evolving connectionist systems, automatic design, bio-inspired techniques, artificial bee colony

## Abstract

One of the essential abilities in animals is to detect novelties within their environment. From the computational point of view, novelty detection consists of finding data that are different in some aspect to the known data. In robotics, researchers have incorporated novelty modules in robots to develop automatic exploration and inspection tasks. The visual sensor is one of the preferred sensors to perform this task. However, there exist problems as illumination changes, occlusion, and scale, among others. Besides, novelty detectors vary their performance depending on the specific application scenario. In this work, we propose a visual novelty detection framework for specific exploration and inspection tasks based on evolved novelty detectors. The system uses deep features to represent the visual information captured by the robots and applies a global optimization technique to design novelty detectors for specific robotics applications. We verified the performance of the proposed system against well-established state-of-the-art methods in a challenging scenario. This scenario was an outdoor environment covering typical problems in computer vision such as illumination changes, occlusion, and geometric transformations. The proposed framework presented high-novelty detection accuracy with competitive or even better results than the baseline methods.

## 1. Introduction

Novelty detection is the task of recognizing data that are different in some aspects from the already known data [1]. This is a challenging problem because the datasets may have a large number of examples of the normal class and an insufficient number of examples of the novel class (in almost all cases, no novelty examples are available). Having robust methods for this type of problem is of great importance in practical applications such as fraud detection [2,3], fault detection [4], medical diagnosis [5,6,7], video surveillance [8,9], and robotic tasks [10,11,12], among others. For these applications, it is not common to have access to data labeled as novel. Another complication is that even when using the same type of information across different applications (e.g., visual information), the concept of novelty varies among them. For these reasons, multi-class classifiers are infeasible for novelty detection. As an alternative, there are dedicated methods for novelty detection that provide all the elements to solve the problem.

In general, the novelty detection methods construct a model with the examples of the normal class and use this model with unknown data to compute novelties. The methods can be classified into five categories [1]: probabilistic, distance-based, reconstruction-based, domain-based, and information-theoretic techniques. One-class classification techniques have been broadly applied for novelty detection with successful results in environments where no dynamic adaptation of the models is required. Recently, advances in deep learning algorithms have shown a new open area into novelty detection [9,13]. The deep-learning-based methods for novelty detection combine the ability of deep neural networks to extract features with the ability of one-class classifiers to model the normal data. The main drawback of these techniques is the need for large-scale datasets and high computational load to train the models.

Inspired by the ability of animals to detect novelties and to respond to changes in their environment [14], researchers have tried to incorporate novelty detection methods into robots to improve their adaptation capability to the dynamic environments that are often present in real-world robotic tasks. Presently, it is possible to capture useful information to perform this process with the use of sensors incorporated into the robots (e.g., sonar, laser, camera, GPS, etc.). Among them, visual sensors are one of the most popular devices to extract information for novelty detection [10,11,15], perhaps because humans use visual information unconsciously as a central component to detect novelties.

In robotics, a novelty detection module is beneficial for several applications (e.g., exploration, inspection, vigilance, etc.). Specifically, in exploration and inspection tasks [11], the robot should explore its environment, building a model of normality using the sensed information. After the model construction, the robot patrols (inspection phase) the same route of the exploration phase in order to detect novelties. It is worth noting that the number of path executions is limited. Although the routes are the same in both phases, due to the operating conditions it is not possible to ensure the same robot positions between different path executions.

For the above problem, the robot needs online novelty detectors to cope with dynamic environments and approaches with fast learning capabilities to detect novelties in scenarios with a reduced amount of information. Most of the traditional one-class classifiers operate offline, which means that it is difficult to adapt these methods to dynamic environments. Meanwhile, deep-learning approaches need large-scale datasets and a huge computation load to train the models. Alternatively, online approaches are based on evolving connectionist systems [11] and grow when required neural networks [16] meet the above conditions. These methods not only build a model of normality incrementally, but they also adapt the model to dynamic changes of the input data—that is, they can insert new information and forget old information. However, we still see challenges in the application of the online novelty detectors into exploration and inspection tasks based on visual information. First, current robotic applications use low-level visual features that are sensitive to illumination changes, occlusion, or geometric transformations. Some visual features used in robotic applications are RGB histograms [11], color angular indexing [17], GIST descriptor [15], and others. Second, in different exploration and inspection tasks, the robots use the same parameters in the novelty detection module, without considering that the performance of the detector depends on the specific task to be solved. These reasons have restricted the applications of the above online novelty detectors to indoor environments where many conditions have been controlled.

Motivated by the previous issues, in this work we propose the application novelty detectors based on evolutionary connectionist systems and grow when required neural networks with visual descriptions drawn from deep convolutional networks for exploration and visual inspection tasks. In contrast with existing deep learning approaches for novelty detection, we propose the use of already-trained networks to extract visual features, instead of learning new visual features, in order to reduce the computational load in the feature extraction phase. We prefer deep descriptions over traditional visual description due to its reliability in generating robust features for classification tasks. Additionally, we propose a framework to design novelty detectors automatically via the selection of the best parameters, depending on the specific robotic exploration and inspection task. This framework uses a global optimization technique as the main component to find the most appropriate parameters for the task. We verified the utility of the proposed visual novelty detection system in outdoor applications, where an unmanned aerial vehicle (UAV) captured images in challenging environments (i.e., environments with illumination changes, geometric transformations in the objects of the environment, and occlusions). In summary, this proposed work presents the following contributions:

1. We extend the application of the above online novelty detectors to outdoor environments where illumination changes, occlusions, and geometric transformations are presented.
2. Most of the existing visual novelty detectors involve humans to select the appropriate parameters for a specific visual exploration and inspection task. In contrast to these previous works, we propose a framework for the automatic design of novelty detectors.
3. In contrast with previous deep-learning and one-class classifiers, our proposal uses a pre-trained convolutional neural network to extract features from images to reduce the computational load. That enables the system to operate online (sample rate of 4 Hz).
4. As far as we know, this is the first time that online novelty detectors based on evolving connectionist systems or grow-when-required neural networks have been applied in unmanned aerial vehicles for detecting novelties in visual exploration and inspection tasks.

The rest of this document is structured as follows. Section 2 reviews some works related to visual novelty detection in robotics. Section 3 presents our visual-based novelty detection approach. Section 4 describes the experimental setup and compares our experimental results against traditional visual novelty detectors. In Section 5, we discuss the results and limitations of this work. Finally, in Section 6 we share our main conclusions and perspectives for future work.

## 2. Related Work

Marsland et al. [14] proposed a self-organizing map (SOM) with a habituation model embedded into the nodes to detect novelty. The system uses sonar readings as inputs, and the nodes habituate to similar inputs. The habituation level of the nodes represents the novelty value of the input. Crook and Hayes [18] developed a novelty detection system based on the Hopfield network—a type of fully-connected recurrent neural network. They implemented the novelty detector in a robot to detect cards in a gallery. The robot captures a color image and through simple processing finds the orange cards. The binary image (detection of the orange color) enters the network to perform the novelty detection process. The operation of the detector consists of updating the weights of the network every time a new input is fed into the network. The system uses a threshold value and the energy level of the network to decide if the input is novel.

Both detectors have restrictions in their operation because they keep a fixed network structure. Therefore, they cannot adapt their behaviors to dynamic changes in the inputs. For this reason, Marsland et al. [16] proposed a novelty detection system for mobile robots based on a grow-when-required (GWR) neural network. The GWR network topologically connects nodes subject to habituation and incorporates new nodes based on their habituation level and the activation level of the nearest node to the given input. Besides, the GWR network can forget patterns, deleting nodes without topological connections. Crook et al. [19] compared the Hopfield-based novelty detector against the GWR network for novelty detection. In this study, they performed two experiments: the first experiment used sonar readings as input, and the second one used images (the problem of card detection in galleries). The results showed that both approaches could construct an appropriate model of the environment. However, the GWR-based approach produced more precise models because of its lower sensitivity to noise, more flexible representation of the inputs, and ability to adapt to dynamic changes in the inputs.

Afterwards, Neto et al. [20] applied a GWR network with visual information as input. They proposed a framework that combines a visual attention model and a visual description of the more salient points in the image based on color angular indexing and the standard deviation of the intensity. This type of description is invariant to illumination changes; however, it is infeasible to detect new objects outside the attention regions. Neto and Nehmzow [17] used the novelty detectors based on GWR and incremental principal component analysis (IPCA) with two interest point detectors: the detection based on saliency and the Harris detector. They compared two ways of representing the patches in the visual input (raw pixels of the image). The first method was to keep a fixed size of the patch, while the second was to find the size of the patch automatically. The results often showed that the fixed-size approach presented the best results. Inspired by the evolving connectionist systems [21] and the habituation model proposed in the GWR networks, Özbielge [11] proposed a recurrent neural network for novelty detection for exploration and inspection tasks. This method predicts the next input and computes a novelty threshold value during its operation. This information is used and compared to the observed input to decide if it is novel. The system uses laser readings, motor outputs, and RGB color histograms as input information. Also, Özbielge [22] proposed a dynamic neural network for static and dynamic environments. The method computes the novelty in a similar way to the previous approach—it computes the error between the input observation and the prediction of the network, and if the error is higher than the evolved threshold, then the object is considered a novelty.

Apart from the above detectors, Kato et al. [15] implemented a system based on reconstruction that takes advantage of the position where the robot captured the images. The novelty detector used the GIST descriptor and a reconstruction-based approach to generate a system invariant to illumination changes. A principal limitation of their system is the absence of a threshold value to detect novelties (no optimization is provided for tuning the threshold). Gonzalez-Pacheco et al. [23] developed a novelty filter to detect new human poses. The system uses visual information of the Kinect sensor and four one-class classifiers: Gaussian mixture model, K-means, one-class support vector machines, and lLeast suares anomaly detection. For this task, the Gaussian mixture model performed better than the other novelty detectors. However, the performance of the method depended on the number of specified Gaussians (the user defined this value in the experiment). Recently, Gatsoulis and McGinnity [24] proposed an online expandable neural network similar to the GWR network. The method uses speeded-up robust features (SURF) and an ownership vector. The main difference between the GRW approach and this method is that the habituation is defined by the object and not by the feature vectors.

All the above novelty detectors have been applied for indoor environments, and few works have been proposed for outdoor environments. For instance, Wang et al. [25] implemented an approximation to the nearest neighbor via search trees to detect novelties in indoor and outdoor environments (they used a static camera for the outdoor environment). The inputs were visual features extracted from patches—for example, color histograms in the HSV space (hue, saturation, value) and texture information (Gabor filters). They compared the performance of their system against the GWR network. The results showed that their proposed approach was better than the GWR network in their particular experiments. Ross et al. [12] presented a vision system for obstacle detection based on novelty for field robotics. The motivation in the use of novelty is that in agricultural applications, it is infeasible to train a system with all types of obstacles. The inputs of the detector were color, texture, and position of the patches in stereo images. The system detects novelty by using the probability density estimated by a weighted version of Parzen windows.

Previous works have explored low-level visual features for image description such as color angular indexing, GIST descriptor, RGB raw values, RGB color histograms, HSV histograms, and Gabor filters, among others. Few efforts have been made to take advantage of emerging deep convolutional neural networks for feature description in visual novelty detection. One such effort is the robotic system proposed by Ritcher and Roy [26]. The objective of this work was to develop a robot with a safe navigation module. An autoencoder network composes the novelty detection module with three hidden layers that automatically find a compressed representation of the image captured by the robot. The goal of the network is to reconstruct the input image, and if the input image cannot be reconstructed (i.e., the error between the input and the output is higher than an error tolerance) then the system will detect the novelty and use it to maintain the safety of the robot.

In summary, most of the existing visual novelty detectors have been configured manually by humans, or no specific procedure for the configuration of the detector has been provided. Also, most of the visual novelty detectors use traditional feature extraction techniques. There are few explorations applying the recent advances in convolutional neural networks as visual feature descriptors. Both the lack of automatic configuration of novelty detectors and the use of low-level traditional visual features have restricted the exploration and inspection task for indoor environments, with controlled conditions (e.g., illumination), and with simple visual novelty detection problems (i.e., conspicuous objects). The proposed work presents an approach to addresses these issues.

## 3. Materials and Methods

**Figure 1:**
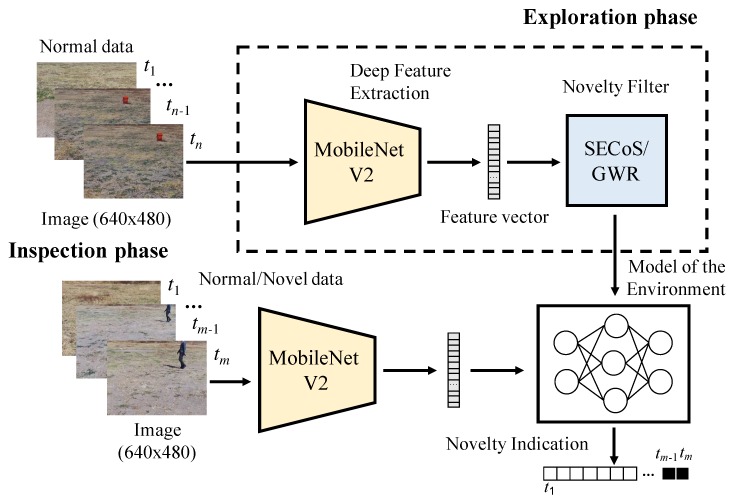
Graphical description of the proposed system for visual exploration and inspection tasks. SECoS: simple evolving connectionist systems.

In this section, we describe the proposed system for visual exploration and inspection tasks. In this work, we used images captured by a UAV operating in outdoor environments. Figure 1 illustrates the proposed system. In the exploration phase, the UAV follows a fixed trajectory and captures images of the environment. The system represents the captured images via deep features by using a pre-trained convolutional neural network called MobileNetV2 [27]. The novelty detector processes the feature vector and constructs a model of the environment. The user can select between two detectors: simple evolving connectionist systems (SECoS) or GWR network. Finally, in the inspection phase, the UAV again executes its path and searches for novel objects. The UAV uses the above model to identify novelties. Then, we describe in more detail the components of the proposed visual novelty detection system.

### 3.1. Visual Feature Extraction

One way to represent the images is via visual feature vectors. Among the visual features, traditional features such as RGB color histograms [11], color angular indexing [10], and the GIST descriptor [15] have been applied for visual novelty detection in robotics. However, traditional visual features are highly sensitive to illumination changes, noise, occlusion, or geometric transformations. Recently, convolutional neural networks have been applied successfully as powerful tools to extract features from images [28], having robust performances in a wide variety of classification tasks.

Motivated by the success of convolutional neural networks as feature extraction methods, we propose the application of a convolutional neural network to extract features from images for the task of visual novelty detection in robotics. In this work, we selected MobileNetV2 [27] because it is the network with the lowest number of parameters in the Keras API and the TensorFlow engine. In our implementation, we used a pre-trained network with the weights trained on the ImageNet dataset. In order to extract the visual features, we resized the input image to the default size in the Keras API of 224×224 pixels. We also deactivated the classification layer and activated the average pooling mode for feature extraction. We obtained visual feature vectors of 1280 elements.

### 3.2. Novelty Detectors

We selected two online novelty detection methods that are used as the base to develop exploration and inspection tasks with real robots [10,11,16]. Both techniques are constructive and can evolve the structures of the models and their parameters during their operation. We selected the SECoS and the GWR network.

#### 3.2.1. Simple Evolving Connectionist Systems

The evolving connectionist systems (ECoS) proposed by Kasabov [21] are a type of neural network that can evolve their parameters and their structure over time. Below, we show the characteristics of the ECoS that make them attractive to address the problem of visual novelty detection in robotics [29]:

- Fast learning capabilities (one-pass learning).
- Online learning and incremental adaptation to new data.
- The model is evolved to adapt to the input information, and the examples are added to the model when they are different in some aspects from the current model of the data.

**Figure 2:**
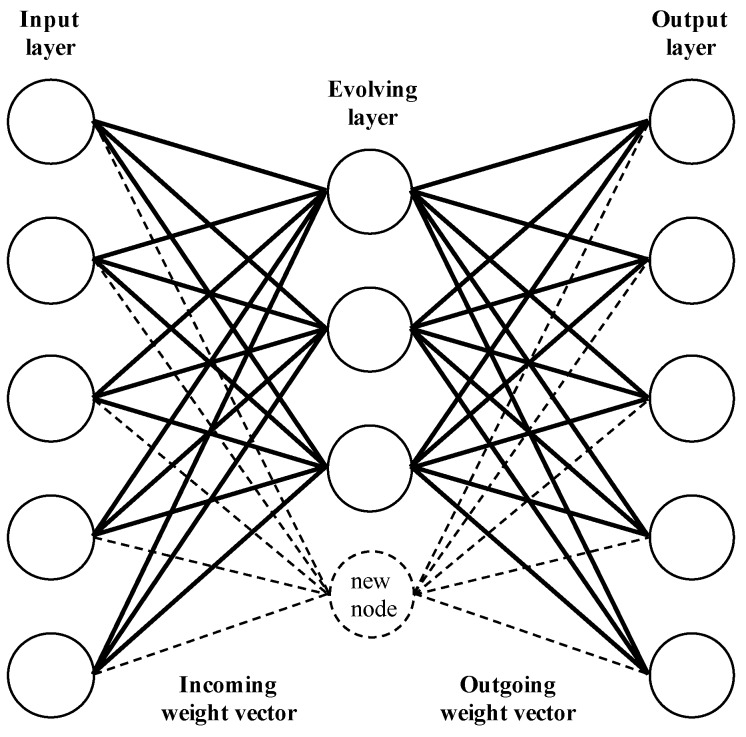
Graphical description of the SECoS network. Adaptation of the general ECoS representation from Watts [29].

The SECoS conserve these characteristics [30], but they present two advantages concerning the other ECoS implementations. The SECoS are easy to implement because they have a low number of layers to learn the input data, and they work directly on the input space. Figure 2 shows a graphical description of the SECoS network. Three layers compose the network: the input layer, which transfers the inputs to the nodes of the next layer; the hidden layer (evolving layer), which incorporates new nodes to represent novel data; and the output layer, which uses saturation linear activation functions to compute the output. In a SECoS network, there are two connection layers: the connections between the nodes of the input layer and the nodes of the evolving layer (incoming connections), and the connections between the nodes of the evolving layer and the nodes of the output layer (outgoing connections).

In this work, we used the SECoS learning algorithm proposed by Watts and Kasabov [30]. The algorithm receives as input the weights of the connections in the network, the input features, and the desired output. The proposed approach uses a SECoS implementation with the same number of nodes in the input layer and the output layer. The objective of the approach is to generate a system able to reconstruct the input vector. When the model generated by the SECoS implementation is not able to represent an input, it should add a new node in the evolving layer with the incoming weight values equal to the input vector and the outgoing weight values equal to the desired output. Also, it should add a new node to the model when the reconstructed output is significantly different from the desired output, that is, when the Euclidean distance between the desired output and the current output of the network is greater than the threshold $E_{thr}$. When the model can represent a given input successfully, the SECoS implementation only updates the model (updating of the connection weights) to better represent the input data. The parameters of this learning model include the learning coefficients ($\eta_{1}$, $\eta_{2}$), the sensitivity threshold ($S_{\mathrm{thr}}$), and the error threshold ($E_{\mathrm{thr}}$). For more details about this learning algorithm, the readers can refer to the work by Watts and Kasabov [30].

#### 3.2.2. Grow-When-Required Neural Network

**Figure 3:**
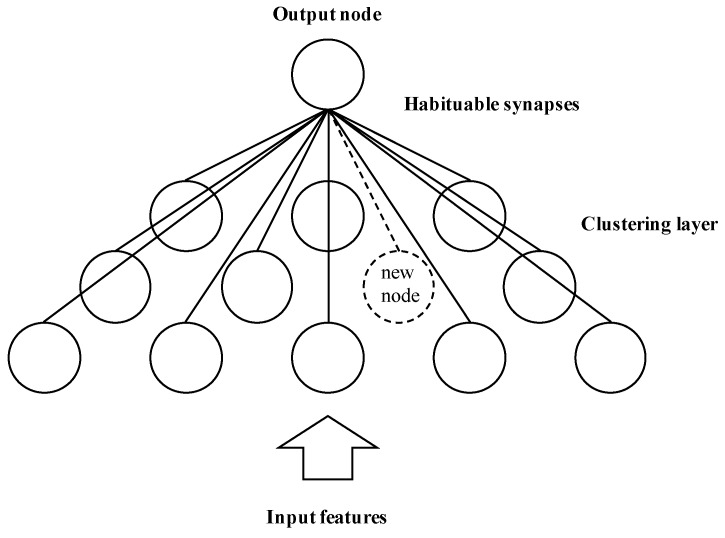
Graphical representation of the grow-when-required (GWR) neural network. Adaptation of the network architecture presented by Neto et al. [20].

GWR is an online self-organized neural network proposed to solve the novelty detection problem [31]. Figure 3 shows a graphical representation of the GWR neural network. A clustering layer of nodes and a single output node compose the network. The nodes in the clustering layer use weight vectors to represent the centers of the clusters. The GWR network can add and remove nodes to its structure, specifically in the clustering layer, to adapt to the changes of the inputs. The connection synapses to the clustering layer in the network are subject to a habituation model, which is a reduction in response to similar inputs.

In the proposed framework, we use the algorithm of the GWR network for novelty detection as described by Neto [10]. The network starts with two dishabituated nodes with weight vectors initialized to the positions of the first two input vectors. At the beginning, there are no topological connections between both nodes. From the third input vector, the best matching node *s* and the second best matching node *t* of the clustering layer are found (i.e., the nearest nodes to the input vector). If there is a topological connection between both nodes, its age is set to zero; otherwise, the connection between both nodes is created with age zero. The GWR network uses the activation and habituation levels of node *s* to decide if the input is novel or not. If the input vector is novel, a new node in the clustering layer is created with its weight vector initialized to the average position between the input vector and the best matching node. Also, the topological connections of the nodes in the clustering layer are updated by removing the connection between the best matching nodes and inserting new connections between the best matching nodes and the created node. Then, the best matching and its topological neighbors update their positions in the direction of the input vector and also update their habituation levels. Finally, all the connections increase their ages and all connections with ages higher than the maximum age are removed. A node is also removed when it has no topological connections (i.e., ability to forget). The parameters that impact the behavior of the network are the parameters of the habituation model, the activation threshold ($a_{T}$), the habituation threshold ($h_{T}$), the proportionality factor (η), and the learning rate (ϵ). A detailed description of the learning algorithm of the GWR neural network can be found in [10].

### 3.3. Global Optimization of Novelty Detectors

**Figure 4:**
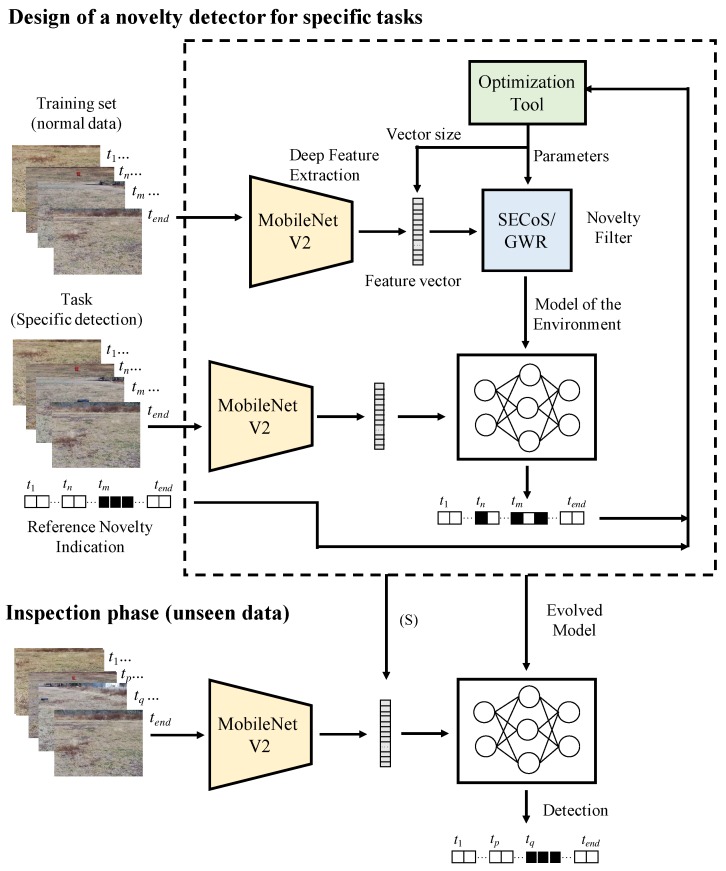
Flowchart of the visual novelty detection for specific tasks. In the training phase, the novelty filter learns to detect a specific object. In the inspection phase, the evolved model is used to detect the object(s) in the environment.

One of the main problems in the application of novelty detectors is the proper selection of their parameters in order to obtain the best results regarding the detection accuracy. With this in mind, we propose a framework to tune the novelty detectors automatically for a specific task (see Figure 4). Our optimization approach not only searches for parameters of the novelty detectors, but also finds the best size of the visual feature vector.

In this work, we propose the use of the artificial bee colony algorithm (ABC) [32] as the optimization tool. Note that although in this work we show the use of the ABC algorithm, in the proposed framework we can incorporate different algorithms to find the more appropriate parameters of the filters to solve specific tasks. The ABC algorithm offers a population-based approach for numerical optimization. In the ABC algorithm, artificial bees update their position over time to find the best food sources. This algorithm has shown to be better than or competitive to other bio-inspired optimization techniques. Besides, we can find applications of the ABC algorithm for a wide variety of engineering problems, such as image processing, data mining, control, and mobile robotics [32]. The implementation details of the algorithm can be found in Mernik et al. [33]. In the proposed methodology, we use an implementation with a termination condition based on the number of iterations, also known as ABC${}_{\mathrm{imp}1}$.

In our implementation of the ABC algorithm, each food position represents a set of parameter values of the novelty detector. Table 1 shows the parameters that should be adjusted by using the ABC algorithm. The search range of all the decision variables is within [0,1]. In the case of the GWR novelty filter, we set the parameters of the habituation model to the default values, and we also keep the maximum age value constant. For the ABC algorithm, we used a population of 20 food positions and a total number of 100 iterations.

## 4. Experimental Preparation

We validated the performance of the proposed method using images captured by a real robot in outdoor environments. We constructed the datasets using these images to train and test the novelty-detection system. We designed an experiment to compare the deep visual feature extraction technique against commonly used visual features for the problem of visual exploration and inspection. In this section, we describe the datasets, the methods for comparison, the experimental setup, and the evaluation metrics.

### 4.1. Datasets

**Figure 5:**
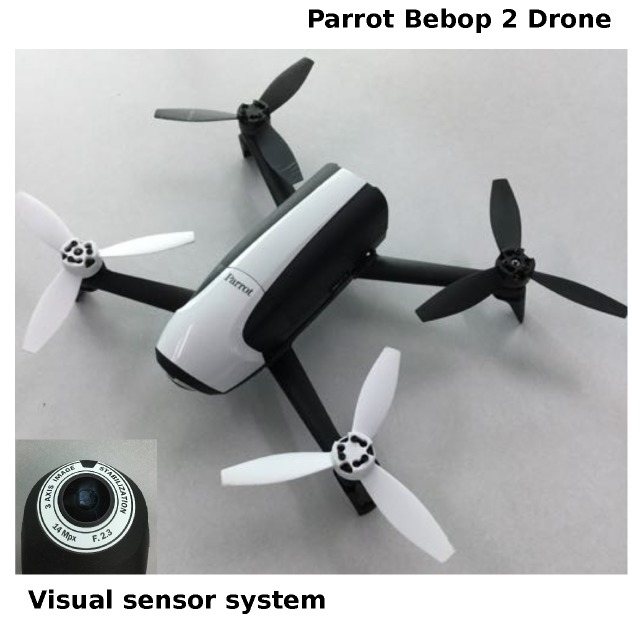
Parrot Bebop 2 Drone with a 14-Mpx flight camera. In the bottom-left corner, we show its visual sensor system.

In this work, we constructed a dataset with images captured by the visual sensor of a UAV. For this purpose, we used a Parrot Bebop 2 Drone with a 14-Mpx flight camera. The captured images had a dimension of 1920 × 1080 pixels, but we constrained the search in the center region of the images with a reduced field-of-view of 640 × 480 pixels. Figure 5 shows the UAV used for data acquisition. Note that the novelty detector system received images of the environment every 250 ms.

**Figure 6:**
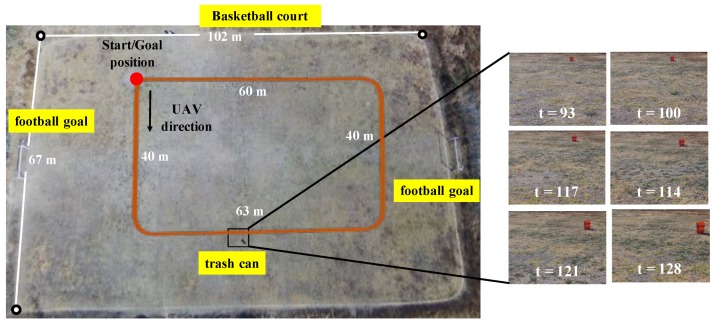
Experimental setup: the outdoor environment, and some sample captured images. UAV: unmanned aerial vehicle.

Figure 6 illustrates the outdoor environment used in this experiment. The UAV executed its default execution control module to fly over the environment in a rectangular shape. In order to generate the datasets, the UAV executed the same path several times with different environment setups.

In the first set of experiments, the UAV flew at 2 m above the ground with morning light conditions (around 11:00 and 12:00). The original environment contained an orange trash can (we called this environment “O-1”). First, the UAV explored the O-1 environment, executing its path two times. The UAV captured a total of 896 images—448 for each execution. Then, it executed the inspection phase and captured another 896 images. In this inspection phase, a person appeared in the environment (we denoted this new environment as O-2). The sequence contains 60 frames with the person. In the second experiment, we added a tire to the O-1 environment (we denoted this environment as O-3). The UAV captured a total of 896 images. The tire is present in 58 frames. Finally, the UAV executed its path in the environment with the person and the tire at the same time. The UAV captured another 896 images in its two path executions. In total, the person is present in 37 frames, and the tire is present in 64 frames. We identified this environment as O-4.

We developed a second set of experiments to test the robustness of the proposed method, considering different scales, types of occlusions, novel objects, and light conditions. In this new set, the UAV flew 4 m above the ground with afternoon light conditions (around 16:00 and 17:00). The methodology to capture the image sequences was similar to the first set of experiments, but with some differences in the settings of the environments. We introduced environment O-5, where the orange trash can was removed. We designed another environment with a person in a different position, and named it O-6. To test the robustness of the proposed method, we added inconspicuous novel objects to environment O-5 (brown boxes). We denoted this environment as O-7. Finally, we set a new environment O-8, where the UAV could visualize how the person occluded the boxes in the environment.

**Figure 7:**
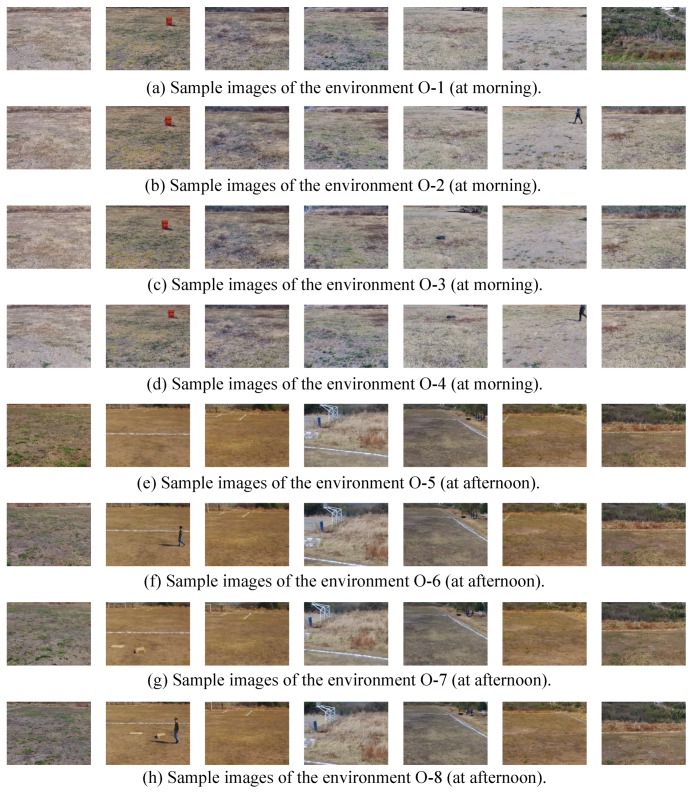
Sample images captured by the UAV in the environments: (**a**) original in the morning (O-1), (**b**) the person in the morning (O-2), (**c**) the tire in the morning (O-3), (**d**) the person and the tire in the morning (O-4), (**e**) empty environment in the afternoon (O-5), (**f**) the person in the afternoon (O-6), (**g**) the boxes in the afternoon (O-7), and (**h**) the person and the boxes in the afternoon (O-8).

Figure 7 shows some sample images of the above environments. Table 2 summarizes the environments used for novelty detection, and Table 3 reports the data partition of the environments to perform the training and test phases.

In all the experiments, the novelty detectors used the images of the training environment of both loops for exploration while only using one loop of the test environment for inspection. The other loop of the test environment was used to evolve the novelty detectors.

### 4.2. Evaluation Metrics

To measure the performance of the novelty detectors, we used the confusion matrix shown in Table 4. TP represents the number of true positives (normal data labeled as normal), TN represents the number of true negatives (novel data labeled as novel), FP represents the number of false positives (novel data labeled as normal), and FN represents the number of false negatives (normal data labeled as novel).

Different metrics have been proposed to reflect the performance reached by the novelty detectors in a single quantity. Three of the most commonly adopted are the $F_{1}$ score, accuracy (ACC), and Matthews correlation coefficient (MCC). Similar to Özbielge [11], we used these three metrics to evaluate the performance of the novelty detectors. These metrics are respectively defined as:

(1) $$F_{1}=\frac{2\cdot \mathrm{TP}}{2\cdot \mathrm{TP}+\mathrm{FP}+\mathrm{FN}},$$

(2) $$\mathrm{ACC}=\frac{\mathrm{TP}+\mathrm{TN}}{\mathrm{TP}+\mathrm{TN}+\mathrm{FP}+\mathrm{FN}},$$

(3) $$\mathrm{MCC}=\frac{\mathrm{TP}\times \mathrm{TN}-\mathrm{FP}\times \mathrm{FN}}{\sqrt{\left(\mathrm{TP}+\mathrm{FP})(\mathrm{TP}+\mathrm{FN})(\mathrm{TN}+\mathrm{FP})(\mathrm{TN}+\mathrm{FN}\right)}}.$$

In the problem of novelty detection, it is important to correctly label all the novel data as novel. Also, it is tolerable to label normal data as novel data, but it is inadmissible to label a novel data as normal. For example, suppose a thief, representing novel data, enters a warehouse. In our novelty detection system, we prefer a system that can detect the thief all the time in order to prevent theft. If the system detects a thief and there is no thief in the scene, there is no problem concerning theft. In order to reflect the desired behavior of novelty detectors, we also incorporated two additional metrics: the true negative rate (*TNR*) and the true positive rate (*TPR*).

To establish the quality of a detector with a single number, we used the average ranking of the measures in all the metrics, inspired by Bianco et al. [34]. Let us consider a set of detectors to be compared, denoted as $M=\{M_{1},M_{2},\ldots ,M_{m}\}$, where *m* is the number of detectors; a set of test images denoted as T; and a set of *P* performance metrics, in this study P=5. We can compute the average ranking of a detector $M_{i}$ as:

(4) $$R_{i}=\frac{1}{P}\sum_{j=1}^{P}\mathrm{rank}\left(M_{i};{\mathrm{measure}}_{j}\left(M_{k}\left(T\right)\right),\;\forall k\neq i\right),$$

where $\mathrm{rank}(M_{i};\cdot )$ computes the rank of the detector $M_{i}$ considering the results of the rest of the detectors in the measure ${\mathrm{measure}}_{j}$.

### 4.3. Experimental Setup

All the algorithms for novelty detection under study can operate online. However, to compare the detectors, they used the same data partition shown in Table 3. We implemented the SECoS, GWR, and ABC algorithms in the C++ programming language. The developed ABC library used the Mersenne Twister pseudo-random generator of 32-bit numbers. In the case of the deep feature extraction technique, we used the pre-trained MobileNetV2 available in the Keras API and the TensorFlow engine. The experiments were developed in a computer with an Intel Core i5 processor, running at 2.9 GHz and with 16 GB of RAM.

To verify the performance of the detectors, we used three traditional visual feature extraction techniques: the RGB color histograms used by Özbilge [11], the color angular indexing used by Neto [10], and the GIST descriptor used by Kato et al. [15]. We compared the performance of the detectors with these feature extraction techniques against the features extracted by the MobileNetV2 network. In this experiment, the system for automatic design used the two image sequences in the exploration phase as training and one sequence of the inspection phase as a validation. The goal of the optimization process was to maximize the performance of the detector concerning the $F_{1}$ score, the ACC, and the MCC. Therefore, we used the following fitness function:

(5) $$f=1-\frac{1}{3}\left(F_{1}+\mathrm{ACC}+\frac{1+\mathrm{MCC}}{2}\right),$$

where f∈[0,1], f=1 represents the worst case with no data classified correctly and f=0 indicates that the novelty detector under study classifies all the data from the validation correctly. In this experiment, we executed 30 simulations for each novelty detector, and we report the average results to perform the comparison.

## 5. Results and Discussion

This section shows and discusses the results of the experiments. We designed the specific novelty detectors for each visual feature independently. We found the most suitable size of the feature vector and the parameters of the novelty detection methods for the particular visual exploration and inspection tasks. In the first part of this section, we compare the results of the proposed feature extraction technique against the well-established feature extraction techniques in the problem of visual novelty detection. Then, we present an analysis of the optimization process of the novelty detectors that use the MobileNetV2 feature extractor. We also show some sample novelty detectors (evolved detectors) generated by the proposed framework and their visual results. Finally, we discuss some limitations of the proposed methodology.

### 5.1. Deep Features and Traditional Visual Features in Novelty Detection

We used well-known visual feature extraction techniques in the problem of novelty detection to compare the performance of the MobileNetV2. We used as reference the RGB color histograms used by Özbilge [11], the color angular indexing applied by Neto [10], and the GIST descriptor implemented by Kato et al. [15]. Table 5 reports the average performance of the novelty detectors in the inspection phase for each dataset, where CAI represents the color angular indexing technique, hRGB represents the RGB color histograms, and MNF represents the feature extraction method based on MobileNetV2. In the table, we also report the average vector size of the features (*VSize*) and the average size of the learned models of the environment (*MSize*)—that is, the average number of nodes in the models. Note that the CAI descriptor produces feature vectors of four elements. In the rest of the descriptors, the optimization process can produce feature vectors of different sizes. In the table, we mark the best-performing method for each metric, according to the specific detector and the particular dataset. The ranking metric uses the *TPR*, *TNR*, $F_{1}$, *ACC*, and *MCC* values to compare the different descriptors for each dataset and detector.

For the D-1 dataset, the objective was to learn a model of the original environment O-1, and to detect a dynamic object represented by a person. In this dataset, the feature extraction technique MNF showed the best performance compared to all other visual extraction techniques. The detectors that used the MNF descriptor could generate compact models of the environment and keep higher performance. They showed accuracies greater than 98%, and *MCC* near 0.9. On the second dataset (D-2), the novelty detectors had to learn a model of the environment O-1 and identify the black tire as the new object. The proposed method achieved the best performance over all others in this dataset—see the ranking of the D-2 dataset in Table 5. The average *ACC* by using both detectors with the MNF technique was around 98%, and the *MCC* was 0.87. Dataset D-3 presents a more challenging situation because the detector was required to learn a model of the environment with a person and detect a black tire. The environment in the inspection phase included both the person and the black tire. Under this situation, the novelty detectors that used the MNF also achieved the best performance, with *ACC* values around 96% for both detectors, and *MCC* values of 0.79 and 0.76 for the SECoS and GWR detectors, respectively. On dataset D-4, the objective was to learn a model of the environment with a tire. In the inspection phase, the person represented the novel object and the black tire represented a normal object. The results indicate that the MNF technique was the second best (the first was the GIST descriptor) with 96% *ACC* and 0.6 *MCC* for both detectors. On dataset D-5, the novelty detectors were required to learn a model of environment O-1 and detect multiple novel objects (both the tire and the person). The MNF description achieved the best performance, with *ACC* values around 97% for both novelty detectors and *MCC* values of 0.89 and 0.88 for the SECoS and GWR detectors, respectively.

On the above datasets, the novelty detectors were tested with novel objects that were highly different from the environment. This could facilitate their detection. In the following, we tested the detectors in more challenging situations. To this end, we used datasets D-6 and D-7, generated by the UAV at a different height (4 m) and with a different light condition (images captured in the afternoon). In the inspection phase of dataset D-6, we used inconspicuous brown boxes to represent the novel objects. In this dataset, the detectors with MNF feature extraction were the best methods to detect novelties, with a ranking of 1.2. Finally, we show the results of the detectors on dataset D-7. The objective in this dataset was to learn a model of an environment with a person and tire and to detect the brown boxes that were occluded by the person in some frames. The results show the superiority of the MNF descriptor for novelty detection, with *MCC* values above of 0.9 and *ACC* values around of 98%, for both detectors.

**Figure 8:**
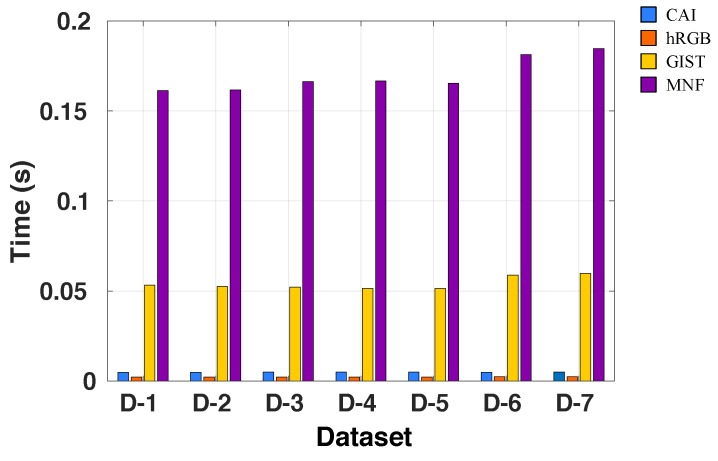
Average time (seconds) to generate the visual features using different descriptors on all datasets.

We then compared the average CPU time to generate the visual features per image on all the datasets. The average time excludes the reading of the image and the post-processing of the visual features. The post-processing only consisted of reducing the vector size to the size found by the optimization process. The reduction was through the average of sectors of equal elements. Figure 8 shows the average time to generate visual features in all the datasets. hRGB was the fastest method, mainly because it only needs to count the number of pixels that belong to a given intensity value. The CAI method was the second fastest method because its computation consists of simple image operations such as average, standard deviation, inverse cosine, and dot product. Meanwhile, the GIST descriptor involves more advanced operations. It includes convolution between the image and Gabor filters at different scales and orientations. The MNF was the slowest feature extraction technique because it includes more complex operations in the image (i.e., it is a deep structure with different convolutional layers). However, all the feature extraction techniques in this work could generate visual features in less than 200 ms—a time that is acceptable for the proposed visual exploration and inspection tasks.

Overall, MNF had balanced results in contrast with the baseline methods. The models found by the MNF descriptor and the novelty detectors were compact, with no more than 35 nodes. In most cases, MNF worked better in detecting novelties than the traditional visual descriptors. Besides, we found that traditional visual features required a low number of nodes to represent the environment. However, their low performance concerning the *ACC* and the *MCC* indicates that the extracted features were insufficient to differentiate the image in the sequences.

### 5.2. Analysis of the Optimization Process

**Figure 9:**
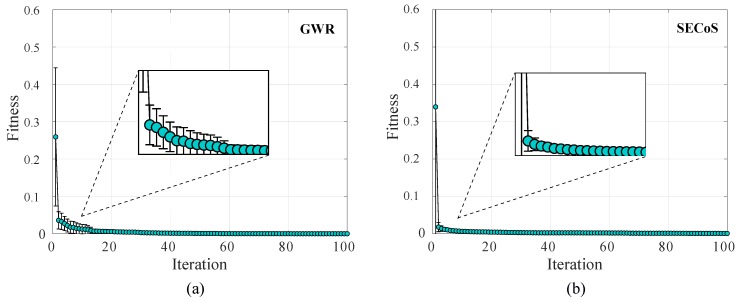
Average fitness value of the best-evolved detectors by using the artificial bee colony (ABC) algorithm in the 30 independent runs on dataset D-2. The detectors used the MNF feature extraction technique: (**a**) GWR detector; (**b**) SECoS detector.

Figure 9 presents the average fitness value of the best-evolved novelty detectors per iteration in the 30 runs on dataset D-2. We will show the optimization processes of both novelty detectors that use the MNF feature extraction technique. In this figure, we also present the standard deviation of the fitness values through bars. At the beginning, the best detectors in the different runs had more variations among them, and this variation was reduced according to the increment in the number of iterations. Analyzing the curve, we can observe that detectors evolved easily on the dataset because they reached fitness values near to the perfect score (zero values), that is, the optimization process found the appropriate parameter values of the detector for the specific novelty detection task. For the GWR, from the initial to the final iteration, it had a decrement of 0.2591 in the average fitness. The more notable change occurred in the first 20 iterations with a change of 0.2532. For the SECoS detector, the optimization process showed a decrease of 0.3386 in the average fitness from the initial to the final iteration. The more significant change occurred in the first 14 iterations, with a change in the average fitness of 0.3341. For the rest of the datasets, the results showed similar behaviors in the optimization process.

**Figure 10:**
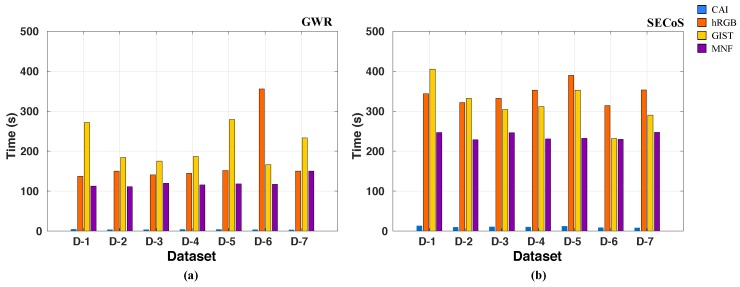
Average CPU time (seconds) to generate a specific novelty detector for each dataset by using different feature extraction techniques: (**a**) GWR detectors; (**b**) SECoS detectors.

Now, we compare the CPU time used in evolving the novelty detectors for specific exploration and inspection tasks of the different feature extraction techniques. Figure 10 shows the average CPU time to evolve the novelty detectors in all the datasets. The search cost excludes the feature extraction phase and includes the post-processing time of the feature vectors. In the figure, we can observe that the GWR detector evolved faster than the SECoS detector. One reason is that the SECoS detectors need to reconstruct the input data and compute the distance to the nearest neighbor node in the novelty detection process, while the GWR method only requires the computation of the distance between the input data and the closest node and the habituation level of this node (without reconstruction).

It is not surprising that the CAI descriptor was the fastest method to evolve the detectors because it keeps the number of inputs in the detector fixed (4 data points) during the entire optimization process. For the rest of the approaches, the vector size varied during the optimization. The maximum number of elements was 778 (3 channels with 256 intensity values), 512, and 256 features for the hRGB, GIST, and MNF descriptors, respectively.

### 5.3. Evolved Novelty Detectors

We used an evolved SECoS detector with deep features on dataset D-3 to illustrate the effects of task-specific novelty detectors. The evolved detector had the following characteristics: $\eta_{1}=0.0183574$, $\eta_{2}=0.4830270$, $A_{\mathrm{thr}}=0.4651190$, $E_{thr}=0.7776980$, and VSize=256. The proposed global optimization process obtained these parameters. In dataset D-3, the training of the detector consisted of generating a model of the O-2 environment (an environment with a person) and the objective was to detect a black tire in an environment with a tire and a person (this new environment was called “O-4”).

**Figure 11:**
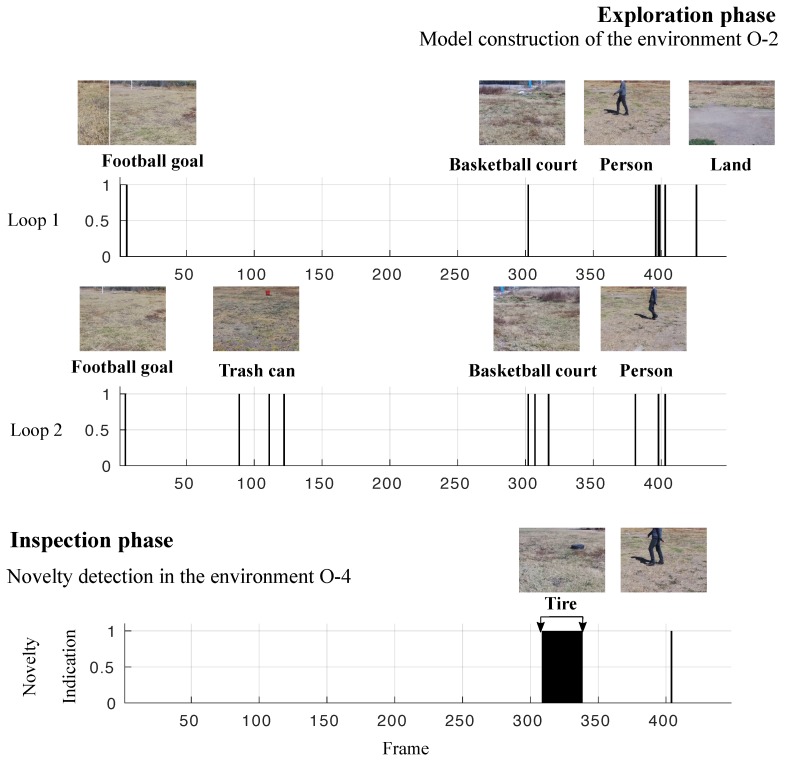
Illustration of the visual exploration and inspection task on dataset D-3 to detect the black tire as the novel object. In the exploration phase, the SECoS detector constructs a model of the environment with the person. In the inspection phase, the detector uses this model to detect the black tire.

Figure 11 presents the exploration and inspection phases by using the evolved SECoS novelty detector. In the exploration phase, the detector constructs the model of the environment finding the most relevant information as the football goal, the orange trash can, the basketball court, and the person. It is commonly adopted for novelty detectors that the first input will be part of the learned model. The image to the left of the football goal in Loop 1 represents the first input image. We used two loops of the same normal environment (O-2) to train the detector. The evolved detector found a model of 18 nodes to represent the O-2 environment. In the inspection phase, the detector uses this model on environment O-4 to detect novelties. In this new environment, the detector found the tire as the novel object in almost all cases, with a single false novelty detection. The performance of this particular detector was *TPR* = 0.9976, *TNR* = 0.9677, $F_{1}$ = 0.9976, *ACC* = 0.9955, and *MCC* = 0.9653.

**Figure 12:**
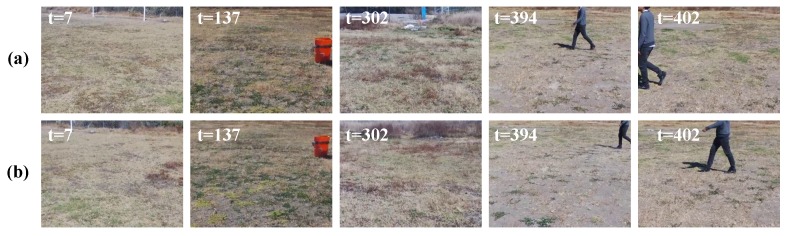
Sample image frames labeled as normal images by the evolved SECoS detector in the inspection phase: (**a**) sample image frames used to learn the model of the environment, and (**b**) sample images detected as normal images in the inspection phase.

**Figure 13:**
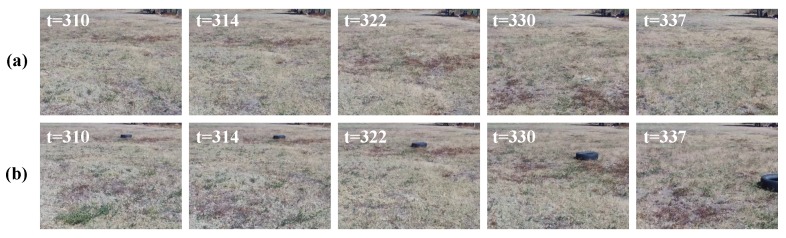
Sample image frames labeled as novelty images by the evolved SECoS detector in the inspection phase: (**a**) sample images frames used to learn the model of the environment, and (**b**) sample images detected as novelty in the inspection phase.

Figure 12 shows some image frames captured by the UAV at different time steps, where the evolved SECoS detector classified these images as normal images in the inspection phase. The first row represents some sample images in the exploration phase, and the second row represents the corresponding image frames in the inspection phase. Although there was considerable variation with the dynamic object and slightly different perspective changes in the images, the evolved detector could classify both situations as part of the normal class. Figure 13 shows some image frames where the evolved SECoS detected novelty: image frames used in the exploration phase at different time steps (see Figure 13a), and some sample images captured in the inspection phase where the detector found the novelty (see Figure 13b). We can observe the black tire at different scale in the images captured in the inspection phases.

Table 6 presents a set of sample novelty detectors generated by the proposed framework for each dataset. We show the parameter values of $\eta_{1}$, $\eta_{2}$, $S_{thr}$, and $E_{thr}$ for the SECoS detectors, and the parameter values of $a_{T}$, $h_{T}$, η, and ϵ for the GWR detectors. The table also reports the found vector size of the deep features for each detector.

In Table 7, we report the performance of the above-evolved detectors. We can observe that the SECoS detectors had similar behavior to the GWR detectors concerning the novelty detection (see the *TNR* values), except on dataset D-5, where the SECOS detector outperformed the GWR. Besides, on datasets D-1, D-3, D-4, D-6, and D-7, the SECoS detectors exceeded the GWR concerning the *TPR* values.

**Figure 14:**
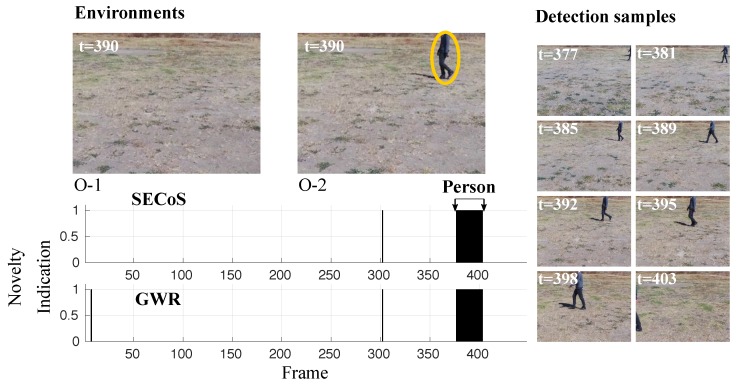
Visual results in novelty detection on dataset D-1, with the person as the novel object.

Now, we introduce some visual results of the evolved detectors in the environments in the morning. In Figure 14, the novelty detectors learned a model of the original environment O-1, and detected the person as the novel object. The figure shows the novelty indication of both methods, an image frame in the exploration phase (picture in the upper left corner), and a picture at the same time step in the inspection phase. We mark the novel object with a yellow ellipse. This figure also presents some successful novelty detections on the right side. From these samples, we can observe the advantage of the evolved detectors, which is that they could detect the person at different scales, perspectives, and occlusion levels.

**Figure 15:**
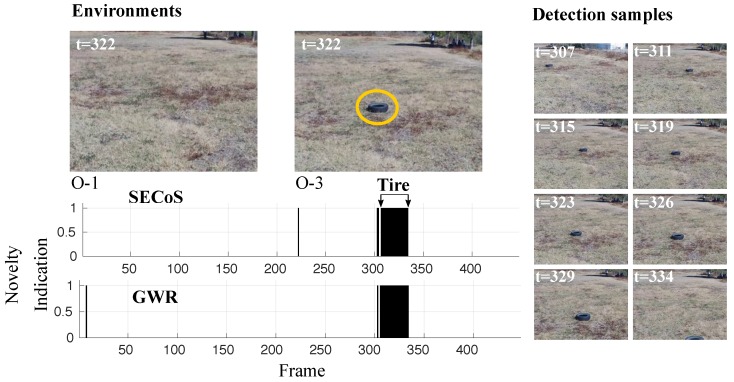
Visual results in novelty detection on dataset D-2, with the tire as the novel object.

Figure 15 shows another example of the visual exploration and inspection task. The task consists of learning a model of the original environment O-1 and to detect the black tire in the inspection phase in environment O-3. The detectors found the tire as the novel object in all cases, the methods could even detect novelties with occlusion; see the last detection sample (t=334), where the tire is almost incomplete.

**Figure 16:**
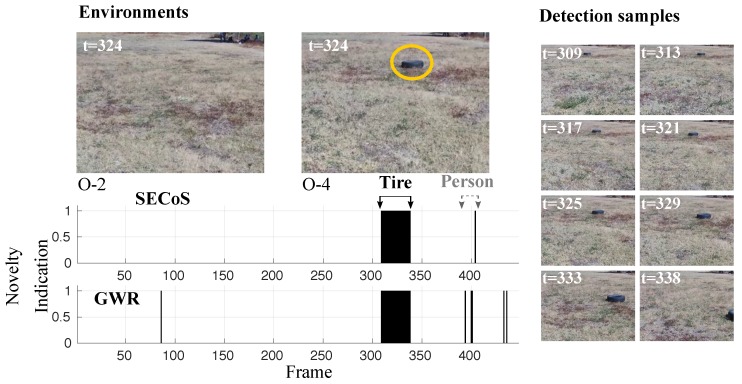
Visual results in novelty detection on dataset D-3 (the tire as the novel object, and the person as the normal object).

**Figure 17:**
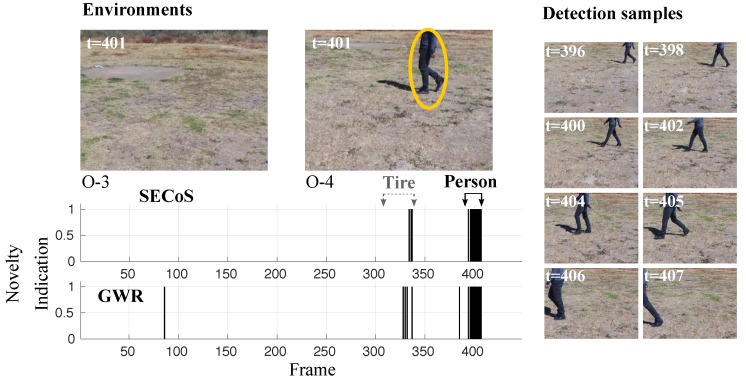
Visual results in novelty detection on dataset D-4 (the person as the novel object and the tire as the normal object).

A more challenging example is presented in Figure 16. In this figure, the detectors should have found that the black tire was the novel object and the person was the normal object. In almost all cases, the methods could detect the novel object. However, some false novelty detections appeared with the person. The SECoS was less sensitive to this phenomenon than the GWR. Another challenging problem is to detect the person as the novel object and the tire as the normal object. Figure 17 illustrates the performance of both detectors in this situation. Like the above example, the methods could detect the person in almost all cases and discover false novelties in the tire.

**Figure 18:**
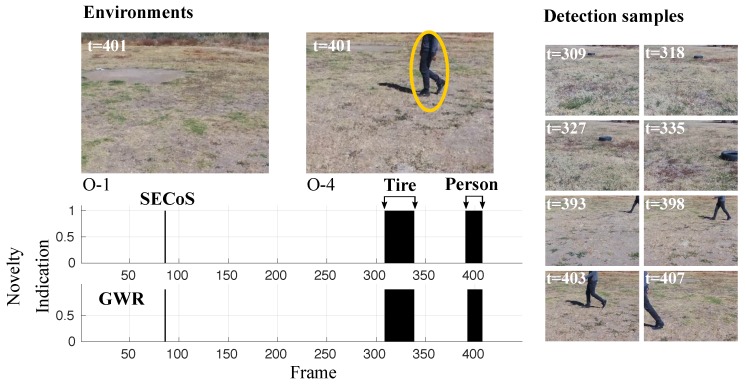
Visual results in novelty detection on dataset D-5, with the person and the tire as the novel objects.

We then present the visual results in detecting both the tire and the person as the novel objects (multiple novel object detection). In this case, both methods could identify the tire and the person with only one false novelty detection; see Figure 18.

**Figure 19:**
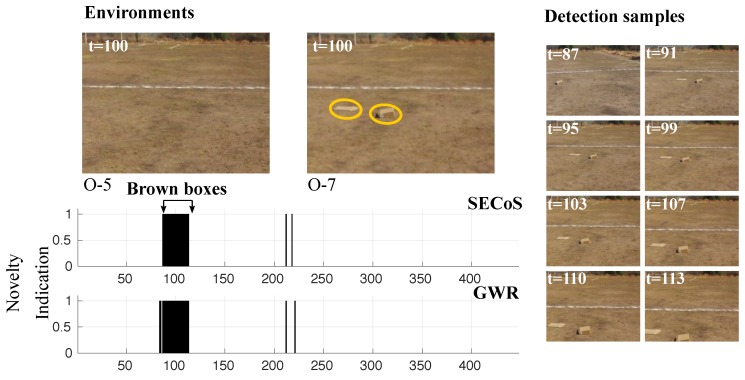
Visual results in novelty detection on dataset D-6 (the brown boxes as the novel objects).

While the previous cases showed results on novel objects that were different from the environment, the next cases show visual exploration and inspection tasks with inconspicuous novel objects (i.e., brown boxes in this experiment). To capture the image frames, the UAV flew at a 4 m height with afternoon light conditions. In Figure 19, the problem was to detect the images with the brown boxes through a learned model of the empty environment in the afternoon (called environment “O-5”). We can observe that the evolved detector detected the brown boxes in almost all cases, with only two false novelty indications.

**Figure 20:**
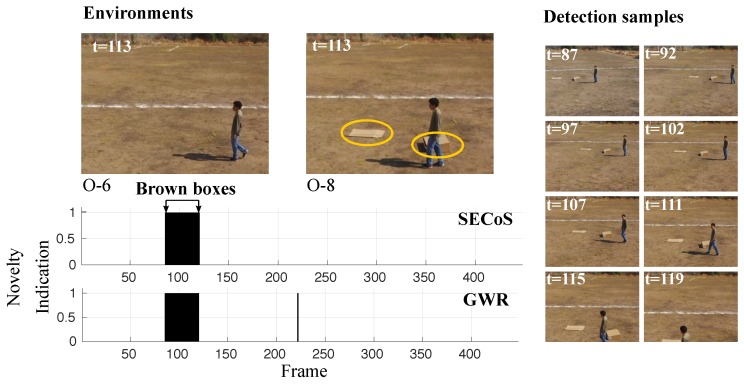
Visual results in novelty detection on dataset D-7 (the brown boxes as the novel objects).

Finally, we show the results of the evolved detectors when a person occluded the brown boxes. Figure 20 presents this situation. The results show that the evolved detectors learned a model of the environment with the person and detected the images with the brown boxes, even if the person occluded them.

In summary, the visual results show that the evolved detectors could identify the novelty in almost all cases. The detectors presented some false novelty detections. However, it is more critical in this type of problem to detect the novelties than to miss the novelties and detect all the normal data. Furthermore, the proposed detectors had excellent capabilities in challenging scenarios with illumination changes, scales, and occlusions.

### 5.4. Limitations

The proposed framework addresses the visual novelty detection in exploration and inspection tasks. Although our proposed method was robust to illumination changes, scale, and occlusion, the evolved detectors presented some issues with abrupt perspective changes in images induced by the flight control of the UAV.

**Figure 21:**
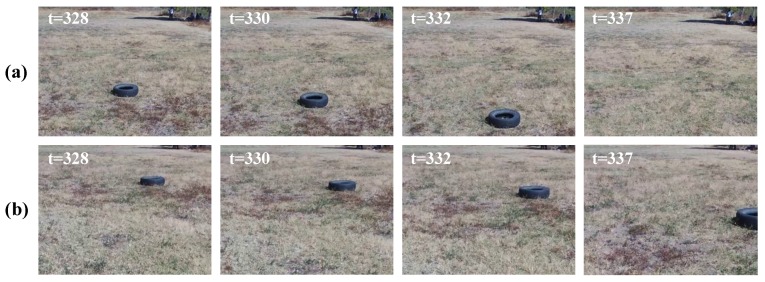
Failure cases in the evolved GWR detector on dataset D-4: (**a**) sample image frames in the exploration phase, and (**b**) false novelty indications in the inspection phase. In the exploration phase, the UAV explores environment O-3. Then, it should detect the person as the novelty in environment O-4. In the inspection phase, due to changes in perspective in the frames induced by the UAV’s flight, some false novelty detections were presented because the information of the frame encoding was too different from the learned model.

Figure 21 shows some failure samples of novelty detections. In the first row, we present some sample images for the training of the evolved novelty detector (GWR in this case). In the second row, we show some sample images in the inspection phase, with a change in the perspective induced by the flight control of the UAV. In the exploration phase, the GWR system builds a model of normality of the environment with the tire (environment O-3). In the inspection phase, the system should detect the person as the novelty in the environment with the tire and the person (environment O-4). Due to the change in perspectives in the image frames in the inspection phase induced by the flight control module of the UAV, these frames were encoded by information that was not currently represented in the learned model of normality. Therefore, the system detected them as novelty. A possible solution to the problem is to evolve the novelty detectors online to adapt to dynamic changes in the environment. Another possible solution is to learn ad-hoc visual features for the problem. We could also explore the incorporation of information from several UAV sensors in order to complement the visual information. With this new information, we could detect new types of novelty, such as novelty based on the object position. All these issues will be the subject of future studies.

## 6. Conclusions

The proposed methodology addresses the problem of automatic design of novelty detectors in visual exploration and inspection tasks, facing the challenge of unbalanced data. We proposed a new framework that uses deep features extracted by a pre-trained neural convolutional network. The methodology exploited the robust capabilities of the deep features to represent the images. A significant contribution of the work is the design of novelty detectors for specific tasks based on a global optimization technique. The proposed methodology simultaneously finds the size of the feature vector and the parameters of the novelty detectors. The methodology was tested on an outdoor environment with images captured by an unmanned aerial vehicle. We considered different types of novelties to verify the performance of the proposed methodology, including conspicuous or inconspicuous novel objects, static or dynamic novel objects, and multiple novel objects. We also considered two different light conditions in the outdoor environment (morning and afternoon), and two different flight heights of 2 m and 4 m, respectively. We performed a comparison with well-established feature extraction techniques in the problem of visual exploration and inspection tasks in the above conditions. The results showed that the proposed methodology is competitive or even better than these traditional techniques. Based on the results, we observed that the evolved detectors are robust to illumination changes, scale changes, and some levels of occlusion. Although they presented some problems with perspective changes produced by the flight control module of the unmanned aerial vehicle, the proposed evolved methods could detect the novelties in almost all cases, which is a desirable characteristic of novelty detection methods.

As future work, we will develop an online technique to design novelty detectors to address dynamic changes in the environment. More studies must be done to test the performance of the methodology with abrupt perspective changes of the objects. Another exciting research direction would be to use sensor fusion to detect novelties when it is difficult to do so with visual information alone.

## Figures and Tables

**Table 1:** Parameters to be tuned for each novelty detector.

Novelty Detector	Parameter	Description
SECoS	$\eta_{1}$	Learning rate 1
	$\eta_{2}$	Learning rate 2
	$S_{\mathrm{thr}}$	Sensitivity threshold
	$E_{\mathrm{thr}}$	Error threshold
GWR	$a_{T}$	Activation threshold
	$h_{T}$	Habituation threshold
	η	Proportionality factor
	ϵ	Learning rate

**Table 2:** Summary of the environments used in the experiments for novelty detection.

Environment	Description	Loops	#Normal	#Novel
O-1	Original setup of the environment (morning).	2	896	0
O-2	A person in the O-1 environment (morning).	2	836	60
O-3	Inclusion of a tire to the O-1 environment (morning).	2	838	58
O-4	A person and tire in the O-1 environment (morning).	2	795	101
O-5	Empty environment (afternoon).	2	896	0
O-6	A person in the O-5 environment (afternoon).	2	822	74
O-7	Inclusion of brown boxes to the O-5 environment (afternoon).	2	835	61
O-8	A person and boxes in the O-5 environment (afternoon).	2	825	71

**Table 3:** Data partition for novelty detection.

Dataset	Exploration	Inspection	Test Case (Novelty)
D-1	O-1	O-2	A dynamic object (person).
D-2	O-1	O-3	A small conspicuous object (black tire).
D-3	O-2	O-4	A conspicuous object in a dynamic environment (black tire).
D-4	O-3	O-4	A dynamic object in an environment with a conspicuous object (person).
D-5	O-1	O-4	Multiple novel objects (person and tire).
D-6	O-5	O-7	Inconspicuous objects (brown boxes).
D-7	O-6	O-8	Occlusion of inconspicuous objects (brown boxes).

**Table 4:** Confusion matrix to evaluate the performance of the novelty detectors. FN: false negative; FP: false positive; TN: true negative; TP: true positive.

Class/Prediction	Normal	Novel
Normal	TP	FN
Novel	FP	TN

**Table 5:** Average results in the inspection phase over the 30 runs. Bold values indicate the best result for each metric according to the specific dataset and the specific novelty detector. CAI: color angular indexing; hRGB: RGB color histogram; MNF: feature extraction based on MobileNetV2; VSize: average vector size; MSize: average model size; TPR: true positive rate; TNR: true negative rate; $F_{1}$: $F_{1}$ score; ACC: accuracy; MCC: Matthews correlation coefficient; *R*: ranking of the detector.

Dataset	Detector	Descriptor	*VSize*	*MSize*	TPR	TNR	$F_{1}$	*ACC*	*MCC*	*R*
D-1	SECoS	CAI	4.0	17.5	0.9692	0.2750	0.9607	0.9258	0.2865	4.0
		hRGB	305.0	12.4	0.9738	0.4571	0.9689	0.9415	0.4673	3.0
		GIST	350.5	47.5	0.9867	0.8571	0.9886	0.9786	0.8312	2.0
		MNF	169.1	7.1	**0.9922**	**0.9000**	**0.9928**	**0.9865**	**0.8859**	**1.0**
	GWR	CAI	4.0	29.1	0.9520	0.3238	0.9532	0.9127	0.2530	3.6
		hRGB	357.3	20.5	0.9757	0.2393	0.9628	0.9297	0.2317	3.4
		GIST	398.1	46.7	**0.9900**	0.8452	0.9898	0.9810	0.8418	1.8
		MNF	153.3	13.8	0.9899	**0.8869**	**0.9912**	**0.9835**	**0.8646**	**1.2**
D-2	SECoS	CAI	4.0	13.6	**0.9879**	0.0155	0.9620	0.9271	0.0076	2.6
		hRGB	337.0	25.3	0.9734	0.0857	0.9567	0.9179	0.0655	3.0
		GIST	384.9	37.7	0.8444	0.8333	0.9084	0.8438	0.4295	3.2
		MNF	143.3	16.6	0.9806	**0.9976**	**0.9901**	**0.9817**	**0.8729**	**1.2**
	GWR	CAI	4.0	2.4	0.9943	0.0000	0.9649	0.9321	−0.0104	3.0
		hRGB	365.4	2.0	**1.0000**	0.0000	0.9677	0.9375	0.0000	2.2
		GIST	334.3	79.8	0.8300	0.7821	0.8976	0.8270	0.3758	3.2
		MNF	180.8	23.7	0.9852	**0.9548**	**0.9910**	**0.9833**	**0.8729**	**1.4**
D-3	SECoS	CAI	4.0	11.8	0.9426	0.6086	0.9561	0.9195	0.4765	2.2
		hRGB	427.2	29.3	0.9642	0.1452	0.9507	0.9075	0.0914	3.2
		GIST	269.0	50.2	0.9019	0.6022	0.9323	0.8812	0.3742	3.6
		MNF	184.1	27.8	**0.9788**	**0.8484**	**0.9836**	**0.9698**	**0.7881**	**1.0**
	GWR	CAI	4.0	6.5	0.9905	0.1118	0.9632	0.9297	0.1111	2.4
		hRGB	445.0	6.1	**0.9922**	0.0118	0.9602	0.9244	0.0024	3.0
		GIST	353.4	152.2	0.9117	0.4645	0.9317	0.8807	0.2852	3.2
		MNF	216.0	34.2	0.9723	**0.8710**	**0.9812**	**0.9653**	**0.7653**	**1.4**
D-4	SECoS	CAI	4.0	16.9	0.9790	0.0157	0.9703	0.9424	−0.0072	3.6
		hRGB	303.2	29.5	0.9745	0.3000	0.9733	0.9489	0.3008	3.0
		GIST	315.0	2.2	**0.9912**	**0.8706**	**0.9930**	**0.9866**	**0.8259**	**1.0**
		MNF	147.2	15.8	0.9729	0.8098	0.9825	0.9667	0.6585	2.4
	GWR	CAI	4.0	6.1	**0.9947**	0.0000	0.9780	0.9570	−0.0105	3.0
		hRGB	289.0	51.9	0.9552	0.3176	0.9633	0.9310	0.2046	3.6
		GIST	334.0	15.3	0.9690	**0.9039**	0.9821	0.9665	**0.7279**	1.8
		MNF	173.8	15.4	0.9770	0.7784	**0.9840**	**0.9695**	0.6578	**1.6**
D-5	SECoS	CAI	4.0	7.5	0.9765	0.0778	0.9356	0.8802	0.0945	3.8
		hRGB	276.5	40.4	**0.9823**	0.1299	0.9414	0.8910	0.1976	2.6
		GIST	306.7	24.6	0.9536	0.5764	0.9512	0.9132	0.5516	2.4
		MNF	180.0	26.9	0.9813	**0.9472**	**0.9874**	**0.9776**	**0.8916**	**1.2**
	GWR	CAI	4.0	7.8	0.9749	0.1049	0.9361	0.8817	0.1565	3.2
		hRGB	331.4	36.6	0.9833	0.0660	0.8978	0.8850	0.0795	3.4
		GIST	385.9	46.7	0.9305	0.6403	0.9420	0.8994	0.5475	2.4
		MNF	221.8	26.9	**0.9917**	**0.8681**	**0.9880**	**0.9784**	**0.8847**	**1.0**
D-6	SECoS	CAI	4.0	7.9	0.9560	0.0344	0.9439	0.8943	−0.0206	3.2
		hRGB	213.2	6.0	0.9270	0.8900	0.9580	0.9246	0.6233	2.4
		GIST	245.4	9.5	0.8352	**0.9167**	0.9059	0.8407	0.4707	3.2
		MNF	150.2	20.4	**0.9750**	0.8911	**0.9834**	**0.9693**	**0.7950**	**1.2**
	GWR	CAI	4.0	11.6	0.9761	0.0200	0.9535	0.9121	−0.0045	2.8
		hRGB	304.3	61.3	0.8946	0.8622	0.9388	0.8924	0.5277	2.8
		GIST	289.0	11.5	0.7977	**0.9111**	0.8825	0.8053	0.4194	3.2
		MNF	210.5	16.0	**0.9796**	0.8878	**0.9857**	**0.9734**	**0.8107**	**1.2**
D-7	SECoS	CAI	4.0	8.5	0.9730	0.0192	0.9487	0.9028	−0.0085	3.8
		hRGB	276.6	6.3	0.9482	**0.9939**	0.9731	0.9516	0.7605	2.8
		GIST	444.9	9.9	**0.9867**	0.9364	0.9907	0.9829	0.8831	2.0
		MNF	165.2	17.6	0.9855	0.9848	**0.9921**	**0.9855**	**0.9065**	**1.4**
	GWR	CAI	4.0	2.8	**0.9982**	0.0000	0.9609	0.9247	−0.0021	3.2
		hRGB	255.7	12.2	0.9369	0.8424	0.9607	0.9300	0.6307	3.4
		GIST	370.7	14.4	0.9654	0.9030	0.9783	0.9608	0.7757	2.2
		MNF	175.7	8.1	0.9862	**0.9960**	**0.9929**	**0.9869**	**0.9162**	**1.2**

**Table 6:** Set of sample evolved detectors generated by the proposed global optimization framework on all the datasets.

Detector	Dataset	$\eta_{1}$	$\eta_{2}$	$A_{\mathrm{thr}}$	$E_{\mathrm{thr}}$	*VSize*
SECoS	D-1	0.2002440	0.2428720	0.0545579	0.4578700	170
	D-2	0.2045570	0.2697980	0.5078360	0.2089900	74
	D-3	0.0183574	0.4830270	0.4651190	0.7776980	256
	D-4	0.1456960	0.3827950	0.1083890	0.2627370	75
	D-5	0.0000000	0.0109682	0.2810780	0.5950580	242
	D-6	0.0000000	0.0000000	0.6285940	0.5863690	144
	D-7	0.6577200	0.2922090	0.1750940	0.4164290	96
		$a_{T}$	$h_{T}$	η	ϵ	*VSize*
GWR	D-1	0.6827340	0.6826510	0.0706664	0.0490785	152
	D-2	0.7888710	0.2963600	0.3931710	0.0000000	101
	D-3	0.5653500	0.3496060	0.4179080	0.0631437	249
	D-4	0.5521850	0.4037900	0.2024040	0.0000000	216
	D-5	0.5756130	0.8404430	0.0000000	0.0000000	256
	D-6	0.7806360	0.7388830	0.2143130	0.0000000	67
	D-7	0.5295850	0.6676220	0.0790152	0.7237070	135

**Table 7:** Results in the inspection phase (unseen data) of the sample evolved detectors. Bold values indicate the best result for each metric.

Dataset	Detector	*MSize*	TPR	TNR	$F_{1}$	*ACC*	*MCC*
D-1	SECoS	6	**0.9976**	0.9643	**0.9976**	**0.9955**	**0.9619**
	GWR	9	0.9952	0.9643	0.9964	0.9933	0.9440
D-2	SECoS	12	0.9929	1.0000	0.9964	0.9933	0.9470
	GWR	27	0.9929	1.0000	0.9964	0.9933	0.9470
D-3	SECoS	18	**0.9976**	0.9677	**0.9976**	**0.9955**	**0.9653**
	GWR	21	0.9856	0.9677	0.9915	0.9843	0.8900
D-4	SECoS	11	**0.9930**	0.7647	**0.9919**	**0.9844**	**0.7802**
	GWR	19	0.9861	0.7647	0.9884	0.9777	0.7118
D-5	SECoS	23	0.9975	**0.9792**	**0.9975**	**0.9955**	**0.9767**
	GWR	32	0.9975	0.9375	0.9950	0.9911	0.9527
D-6	SECoS	17	**0.9952**	0.9000	**0.9940**	**0.9888**	**0.9094**
	GWR	14	0.9904	0.9000	0.9916	0.9844	0.8770
D-7	SECoS	9	**0.9952**	1.0000	**0.9976**	**0.9955**	**0.9687**
	GWR	4	0.9928	1.0000	0.9964	0.9933	0.9540
